# Study on The Effect of Royal Jelly on Reproductive Parameters in Streptozotocin-Induced Diabetic Rats 

**DOI:** 10.22074/ijfs.2015.4215

**Published:** 2015-04-21

**Authors:** Elham Ghanbari, Vahid Nejati, Gholamreza Najafi, Mozafar Khazaei, Mohammad Babaei

**Affiliations:** 1 Department of Biology, Faculty of Science, Urmia University, Urmia, Iran; 2 Department of Basic Science, Faculty of Veterinary Medicine, Urmia University, Urmia, Iran; 3 Fertility and Infertility Research Center, Kermanshah University of Medical Sciences, Kermanshah, Iran

**Keywords:** Diabetes Mellitus, Male Rat, Royal Jelly, Sperm

## Abstract

**Background:**

Diabetes mellitus has a variety of structural and functional effects on the male reproductive system. Diabetes results in reduced sperm parameters and libido. The present study aims to investigate the effects of royal jelly (RJ) on reproductive parameters of testosterone and malondialdehyde (MDA) production in diabetic rats.

**Materials and Methods:**

This experimental study was conducted on adult male Wistar rats. The animals were divided into four groups (n=8 per group): control, RJ, diabetic and diabetic treated with RJ. Diabetes was induced by intraperitoneal injection of 60 mg/kg body weight (BW) of streptozotocin (STZ). RJ, at a dose of 100 mg/kg BW was given by gavage. The duration of treatment was six weeks. After the treatment period the rats were sacrificed. The testes were weighed and changes in sperm count, motility, viability, deformity, DNA integrity and chromatin quality were analyzed. Serum testosterone and MDA concentrations of testicular tissue were determined. Data were analyzed by oneway ANOVA with p<0.05 as the significant level.

**Results:**

STZ-induced diabetes decreased numerous reproductive parameters in rats. Testicular weight, sperm count, motility, viability and serum testosterone levels increased in the diabetic group treated with RJ. There was a significant decrease observed in sperm deformity, DNA integrity, chromatin quality, and tissue MDA levels in diabetic rats treated with RJ compared to the diabetic group (p<0.05).

**Conclusion:**

RJ improved reproductive parameters such as testicular weight, sperm count, viability, motility, deformity, DNA integrity, chromatin quality, serum testosterone and testicular tissue MDA levels in diabetic rats.

## Introduction

One of the major health problems in life is infertility; male factors comprise approximately 30% of this problem (1). Several factors can affect the spermatogenesis process and decrease sperm quality and quantity. Diabetes mellitus, liver and coronary heart diseases, air pollutants, chronic smoking and vitamin deficiency affect spermatogenesis (2). Diabetes leads to vacuolization in Sertoli cells, raises apoptosis in spermatogonia cells and spermatocytes in seminiferous tubules of male rats (3). According to research, male reproductive dysfunctions in animal models of diabetes include decreased semen quality, testicular weight, sperm count and motility, and testosterone levels in addition to increased abnormal sperm and oxidative damage in the testes (4). 

Oxidative stress has been described as an important factor in many diseases such as diabetes (5). Although the precise mechanism for diabetes mellitus is not well understood, increased production of free radicals is the major mechanism that causes damage (6). Diabetes mellitus has an extensive and close association with oxidative stress induced by exacerbation of oxygen free radical formation. Hyperglycemia is associated with increased oxidative stress and leads to many complications in different tissues (7). Evidence indicates that free radicals, membrane lipid peroxidation and protein oxidation are increased in diabetic patients and diabetic animals. The production of reactive oxygen species (ROS) is a normal physiological event in the testes. Excessive production of ROS can be detrimental to sperm, being associated with male infertility. The spermatozoa plasma membrane contains a high amount of unsaturated fatty acids. Therefore, it is susceptible to peroxidative damage (8). The lipid peroxidation destroys the structure of the lipid matrix of spermatozoa membranes and disturbs sperm motility (9). 

Royal jelly (RJ), a food item secreted by the hypopharyngeal glands of worker honeybees is a mixture that contains lipid, glucose, protein, vitamins and minerals. RJ is widely used as a commercial medical product. Previous studies have shown that RJ has many chemical and physical properties such as anti-tumor, antioxidant, anti-inflammatory and immune-modulatory functions in animals (10). Estrogenic activity similar to other exogenous steroid hormones (11), higher testosterone content and intensive spermatogenesis in hamster testes (12), and increased serum testosterone levels in heat-stressed male rabbits are reported for RJ (13). Further, research has shown that alkaline and water extracts of RJ have high scavenging ability and antioxidative activity against active oxygen species (14). 

Another study has shown that RJ collected 24 hours after larval transfer has the strongest antioxidative action (15). Various *in vitro* experimental models on rats have also proven the antioxidative activity of RJ (16) and protection against oxidative stress has been confirmed in experiments on laboratory animals (17). The present study aims to investigate the effect of RJ on reproductive parameters and malondialdehyde (MDA) levels of testicular tissue in diabetic male rats. 

## Materials and Methods

### Animals

In this experimental study, 32 healthy adult male Wistar rats (200±10 g) were used. The animals were obtained from the animal house at the Faculty of Science, Urmia University. The rats were kept under specific conditions on a constant 12-hour light/dark cycle and at a controlled temperature of 21±2˚C. They were fed with standard diet pellets and allowed food and water ad libitum for an acclimation period of one week. The animals were housed in polypropylene cages and maintained in a strictly controlled environment. This study was conducted in accordance with the Guidelines of the Ethical Committee for Research on Laboratory Animals at Urmia University. 

### Experimental design

We randomly divided 32 male Wistar rats into four groups (n=8 per group): control [1 cc of distilled water (DW) day]; RJ (100 mg RJ/kg of BW/ day); diabetic (1 cc of DW/day) and diabetic treated with RJ (100 mg RJ/kg of BW/day). 

Fresh RJ was obtained from a local beekeeping association (Urmia, Iran) and stored at −20 ˚C until use. The RJ was confirmed by an expert academic member (Urmia University). RJ was dissolved in distilled water and administered orally to the RJ and diabetic treated RJ groups for 42 consecutive days. The diabetic and diabetic treated RJ groups were given a single intraperitoneal administration of streptozotocin (STZ, S0130-500 MG, SigmaAldrich Co, St. Louis, MO, USA) at 60 mg/kg BW dissolved in 0.1 M citrate buffer, pH=4.6 (18). At 72 hours after administration, the rats were allowed to fast for 18 hours after which their blood sugar levels were measured by tail puncture using a glucometer (IGM-0002A, GP5EAKFO9548). Rats that had blood sugar levels above 300 mg/ dl were considered to be diabetic and included in this study. 

On the 42^nd^ day of treatment, the rats were sacrificed by intraperitoneal injection of 80 mg ketamine and a laparotomy was conducted. The testes and epididymides were collected. The cauda region of the epididymis was used for evaluation of sperm parameters. The right testes were processed for histopathological studies whereas the left testes were homogenated for biochemical estimations of MDA.

### Sperm count

Epididymal sperm were collected by slicing the cauda region of the epididymis into small pieces in 1 ml human tubal fluid (HTF)+4 mg/ml bovine serum albumin (BSA) and incubated for 30 minutes at 37˚C in 5% CO_2_ to allow the sperm to swim out of the epididymal tubules. Sperm count was performed with a hemocytometer. Results were expressed as millions of sperm/ml. A few drops of the diluted sperm suspension, as a sample, was transferred onto a Neubauer’s improved counting chamber (depth: 0.1 mm) and allowed to remain for 5 minutes (19).

### Sperm morphology

We evaluated sperm morphology by analyzing sperm smears made from the left cauda epididymides. An aliquot of the sample was used for preparation of the smears in order to evaluate deformities to the spermatozoa (18). Eosin/nigrosin stain was used to estimate spermatozoa morphology. To test, one drop of eosin/nigrosin was added to the suspension and mixed gently. The slides were then viewed under a light microscope at ×400 magnification. A total of 300 spermatozoa were analyzed on each slide for abnormalities of the head and tail (19). 

### Sperm viability

To assess sperm viability, 10 μl of eosin/nigrosin was added to an equal volume of spermatozoa suspension. After 2 minutes of incubation at room temperature, slides were viewed at ×400 magnification. Sperms with altered plasma membranes appeared pink and those with intact plasma membranes remained unstained. In each sample, 400 sperm cells were counted and the percentages of sperm viability (ratio of sperm with intact/altered plasma membranes) were calculated (19). 

### Sperm motility

The percentage of sperm motility was evaluated visually by a light microscope (Olympus Co., Tokyo, Japan) at ×400 magnifications. For this process, one drop of sperm suspension was placed on a glass slide which was then covered with a lamella. The number of sperm that had rapid progressive forward movement (RPFM), slow progressive forward movement (SPFM), circumferential motion (CM) and those which remained motionless (ML) were counted in several microscopic fields of vision and the percentages of motile and non-motile sperm were obtained. Motility estimates were obtained from ten different fields in each sample (18). 

### Body and testes weights

Animals were weighed to monitor their general health. The testes and epididymides were removed. The testes were weighed and processed for biochemical analysis. 

### Acridine orange (AO) DNA denaturation assay

The AO test is a simplified microscopic sperm chromatin structure assay which reflects sperm chromatin denaturation. A drop of the sperm suspension was spread on the glass slides and allowed to air-dry. All smears were fixed in methanolacetic acid at 1:3 v/v for 2 hours. The slides were then stained with 2-3 cc of 19% AO solution in phosphate citrate for 5 minutes, then rinsed with deionized water. The sperm were evaluated by a fluorescence microscope (Zeiss Company, Germany). Two types of staining patterns were identifiedgreen (double-stranded DNA) and yellow (singlestranded DNA) sperm (20). 

### Aniline blue (AB) chromatin quality assay

A drop of spermatozoa suspension was spread on glass slides and allowed to air-dry. All smears were fixed in 3% glutaraldehyde in phosphate buffered saline. The slides were then stained with 5% aqueous AB and mixed with 4% acetic acid (pH=3.5) for 5 minutes. Sperm heads that contained immature nuclear chromatin stained blue whereas those with mature nuclei did not stain. The percentage of spermatozoa that stained AB was determined by counting 400 spermatozoa (21). 

### Hormone assay

Blood serum was separated by centrifuge (3000 g for 15 minutes) and serum samples were directly frozen at –70˚C until biochemical analyses. Serum testosterone concentrations were measured by an electrochemiluminescence testosterone kit (Demeditec Diagnostics GmbH, Kiel, Germany). The amount of testosterone was expressed as ng/dL (22). 

### Malondialdehyde (MDA) level assay

Fresh tissue samples were minced and homogenized under ice-cold conditions. The testicular tissues 116 were homogenized into an ice-cold 1.15% solution of KCl to obtain a 10% (w/v) homogenate. Then, 300 µl of 10% trichloroacetic acid (TCA) was added to 150 µl of the homogenized sample and centrifuged at 1000 rpm for 10 minutes at 4˚C. The supernatant was transferred to a test tube with 300 µl of 67% thiobarbituric acid (TBA) and incubated at 100˚C for 25 minutes. After 5 minutes of cooling, a pink color appeared because of the MDA-TBA reaction. Absorbance was evaluated using a spectrophotometer (Pharmacia, Novaspec II, Biochrom, England) at wavelength of 535 nm (23). The level of lipid peroxides was expressed as µmol MDA/mg protein. 

### Statistical analysis

Data were presented as mean±SEM and analyzed by one-way ANOVA followed by the Tukey test using SPSS package (version 18) and p<0.05 as the significance level. 

## Results

### Body weight (BW) and testes weight

The initial BW of the rats did not significantly differ between the groups. A significant difference in the final BW of the rats was observed. The diabetic rats showed reduced BW but diabetic rats treated with RJ had a significant increase in BW (p=0.000, Table 1). 

The testicular weights of the rats are shown in table 1. Diabetes caused a statistically significant decrease in testes weights compared to the control group (p=0.000). The administration of RJ caused an increase in testes weight compared to the control group. The testes/BW ratio revealed a significant decrease in the diabetic group compared to the control group. The group treated with RJ had a significant increase in the testes/BW ratio. However, significant differences were not observed in other groups (p=0.000, Table 1). 

## Sperm parameters

Diabetes caused a significant decrease in sperm count compared with the control group (p=0.000). Treatment with RJ significantly increased cauda epididymal sperm count. However, administration of RJ to treated diabetic rats significantly prevented the STZ-induced negative effects on sperm count compared with the diabetic group (p=0.000, Table 2). 

**Fig.1:**
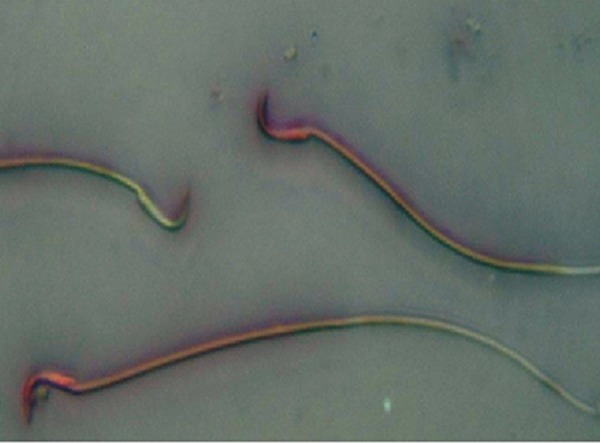
Sperm with altered plasma membranes appeared pink whereas those with intact plasma membranes did not stain (EN ×400).

There was a significantly lower percentage of sperm viability in diabetic rats than those of control and RJ groups (p=0.000). The diabetic group treated with RJ showed a significant increase in percentage of sperm viability in compared with the diabetic group (p=0.000, Table 2, Fig.1). 

The percentage of sperm deformity increased significantly in the diabetic group compared with the control and RJ groups (p=0.000). The diabetic group treated with RJ showed a significant decrease in percentage of deformed sperm compared with the diabetic group (p=0.000, Table 2). 

In terms of sperm motility, RPFM decreased significantly in the diabetic group when compared with the control group (p=0.010). Furthermore, SPFM and ML increased in the diabetic group. Daily administration of RJ caused a significant increase in sperm motility and type of RPFM compared to the diabetic group (p=0.010, Table 3). 

## Acridine orange (AO) DNA denaturation

**Fig.2:**
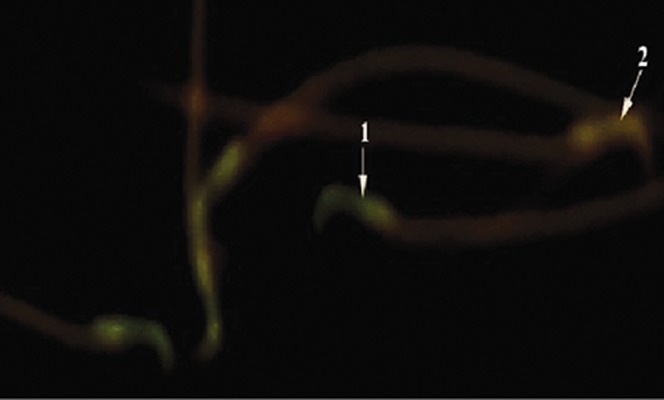
Diabetic treated royal jelly (RJ) group: 1. Sperm with normal DNA integrity showed green fluorescence and 2. Those with DNA damage stained orange-red (AO ×1000). AO; Acridine orange.

We observed a significant increase in the percentage of spermatozoa with DNA damage in the diabetic group compared with the control and RJ groups (p=0.010, Table 4, Fig.2). 

## Aniline blue (AB) chromatin quality

Treatment with RJ showed a markedly significant decrease in the percentage of spermatozoa with chromatin abnormalities (p=0.010, Table 4, Fig.1). 

## Testosterone level

Statistical analysis showed that intraperitoneal administration of STZ to normal rats induced a significant decrease in serum testosterone to 3.93±0.46 ng/dL versus 6.25±0.13 ng/dL in the control group (p=0.000). Oral administration of RJ at 100 mg/kg BW for 42 days to diabetic rats caused a significant increase in serum testosterone levels compared with the diabetic group (p=0.000, Table 5). 

## Malondialdehyde (MDA) levels

Diabetes induced lipid peroxidation in the testis tissue as revealed by a significant rise of MDA in the diabetic group compared with the control and RJ groups (p=0.000). MDA contents in the diabetic treated with RJ group were lower than those in the diabetic group. Therefore, RJ administration caused a partial decline of testis tissue MDA levels in the diabetic group treated with RJ (p=0.000, Table 5). 

**Table 1:** Effect of oral administration of royal jelly (RJ) for 42 days on weight characteristics of male rats (n=8 per group)


Groups	Initial BW (g)	Final BW (g)	Testis weight (g)	Testis/BW ratio (%)

**Control**	194.4±4.91 ^a^	225.33±2.73 ^a^	2.00±0.01 ^a^	0.89±0.01 ^a^
**RJ**	193.9±6.43 ^a^	212.47±4.22 ^a^	2.04±0.08 ^a^	0.96±0.01 ^b^
**Diabetic**	206.7±9.79 ^a^	123.86±6.07 ^b^	0.76±0.07 ^b^	0.61± 0.02 ^c^
**Diabetic/RJ**	192.6±7.40 ^a^	173.93±6.73 ^c^	1.50±0.05 ^c^	0.86±0.01 ^a^


Data are presented as mean±SEM. Values with different letters indicate significant differences among groups at p≤0.05. BW; Body weight.

**Table 2:** Effect of oral administration of royal jelly (RJ) on sperm parameters in male rats (n=8 per group)


Groups	Count (10^6^/ml of suspension)	Viability (%)	Deformity (%)

**Control**	49.70±1.17 ^a^	88.89±0.74 ^a^	3.19±0.12 ^a^
**RJ**	50.08±0.56 ^a^	85.15±3.58 ^a^	3.28±0.17 ^a^
**Diabetic**	30.79±1.11 ^b^	55.48±3.33 ^b^	9.19±0.53 ^b^
**Diabetic/RJ**	49.00±1.77 ^a^	71.44±2.34 ^c^	4.34±0.57 ^a^


Data are presented as mean±SEM. Values with different letters indicate significant differences among groups at p≤0.05.

**Table 3:** Effects (%) of royal jelly (RJ) on sperm motility in male rats (n=8 per group)


Groups	Sperm motility (RPFM)	Sperm motility (SPFM)	Sperm motility (CM)	Sperm motility (ML)

**Control**	63.25±1.69 ^a^	17.15±0.64 ^a^	11.20±0.77 ^a^	7.43±0.45 ^a^
**RJ**	64.73±0.71 ^a^	17.81±0.81 ^a^	11.72±0.69 ^a^	10.02±0.67 ^a^
**Diabetic**	47.88±6.01 ^b^	23.57±0.79 ^b^	10.56±0.83 ^a^	10.89±1.16 ^b^
**Diabetic/RJ**	59.36±0.98 ^a^	15.55±0.26 ^a^	13.88±0.97 ^a^	8.95±0.51 ^a^


Data are presented as mean±SEM. Values with different letters indicate significant differences among groups at p≤0.05. RPFM; Rapid progressive forward movement, SPFM; Slow progressive forward movement, CM; Circumferential movement and ML; Motionless.

**Table 4:** Effect of royal jelly (RJ) on DNA damage and chromatin abnormalities of sperm in male rats (n=8 per group)


Groups	AB^+^ (%)	AO^+^ (%)

**Control**	10.33±0.88 ^a^	9.67±1.45 ^a^
**RJ**	10.67±0.89 ^a^	10.33±0.88 ^a^
**Diabetic**	23.33±2.03 ^b^	22.67±1.20 ^b^
**Diabetic/RJ**	14.67±1.20 ^a^	14.67±2.03 ^a^


Data are presented as mean±SEM. Values with different letters indicate significant differences among groups at p≤0.05. AB; Aniline blue and AO; Acridine orange.

**Table 5:** Effect of oral administration of royal jelly (RJ) for 42 days on serum testosterone hormone and malondialdehyde (MDA) levels in male rats (n=8 per group)


Groups	Testosterone (ng/dL)	MDA (µmol/g tissue)

**Control**	6.25±0.13 ^a^	455.13± 7.40 ^a^
**RJ**	7.13±0.24 ^a^	445.18± 4.73 ^a^
**Diabetic**	3.93±0.46 ^b^	660.01± 12.61 ^b^
**Diabetic/RJ**	6.32±0.09 ^a^	524.30± 19.96 ^c^


Data are presented as mean±SEM. Values with different letters indicate significant differences among groups at p≤0.05.

## Discussion

The current study sought to determine the effect of RJ on reproductive parameters of diabetic male rats. We observed increased testicular weight, sperm count, sperm motility, number of viable spermatozoa and serum testosterone levels and decreased MDA level of testes tissue in the diabetic group treated with RJ. To our knowledge, this was the first report on the effect of RJ on diabetic male rats.

Diabetes decreased testicular weight and seminal vesicles, induced male reproductive dysfunctions, decreased serum testosterone levels and lowered semen quality and quantity. It is well-known that diabetes is positively associated with lowered male fertility and sexual disturbances (2). Previous studies have indicated that the neuropathy and vascular insufficiency caused by diabetes may be related to sexual dysfunction (24). Male sexual dysfunction in STZ-induced diabetic rats results from the alterations of the pituitary–testicular tract axis (25). Our results have clearly confirmed a decrease in testicular weight and serum testosterone levels in STZ-induced diabetic rats.

It has been demonstrated that oral administration of RJ improves the physiological status and a series of sperm parameters in heat-stressed male rabbits (13). Our study also showed that oral administration of 100 mg/kg BW of RJ to diabetic male rats for 42 days caused an increase in testes weight, viable sperm percentage, serum testosterone level and sperm motility, and decreased the number of abnormal sperm in diabetic rats. These results were attributed to the improvement of reproductive parameters in diabetic male rats by RJ, which was attributed to its antioxidant and estrogenic activities.

Diabetes is considered an important endocrine disease in the metabolism of carbohydrates. These changes result in an increase in free radical formation and LDL-oxidase production (6). Previous study have reported that diabetes causes a marked oxidative impact as evidenced by the significant increase in testicular lipid peroxidation as well as a significant decrease in testicular antioxidants, including CAT and GSH-R activities and GSH content (26). A number of studies have reported that intake of antioxidants and vitamins A, B, C, and E can increase stability of the testicular blood barrier and protect sperm DNA from oxidative stress caused by active free radicals (27).

RJ contains vitamins E and C (14) which have been reported to increase glutathione followed by decreased MDA levels in adult male rats (28). Vitamins E and C, whose antioxidant roles have been proven and shown to inhibit free radicals, induced damage to sensitive cell membranes of the testis and reduced lipid peroxidation in tissue estimation by MDA. Vitamins E and C significantly decreased MDA levels and increased glutathione levels (29). This study also showed that MDA levels in the testes decreased in diabetic rats treated with RJ. This effect could be attributed to vitamins C and E in RJ.

Studies have shown a direct link between blood sugar levels and sperm quality. In patients with high blood sugar levels, the incidence of non-viable sperm in seminal plasma is higher (6). Batchelder reported that RJ decreased blood sugar levels via the insulin-like material and other compounds (vitamins B3 and H and chromium). Also it has been noted that RJ is capable of sustaining a high level of blood sugar by participating in the oxidation of glucose to procure energy via the insulin-like material found in RJ. Furthermore, the insulin found in RJ highly resembles the insulin found in mammals (30). Therefore, our results have shown that RJ improved reproductive parameters and decreased MDA levels in the testicular tissue of diabetic rats.

## Conclusion

Oral administration of RJ to diabetic male rats decreased sperm deformity, DNA damage, chromatin abnormalities and testicular tissue MDA levels and increased testicular weight, sperm count, motility and viability. Therefore, this study has suggested that intake of RJ may be useful for diabetic patients who suffer from sexual impotency, as RJ produces anti-diabetic activity and exhibits fertility enhancing properties in male diabetic rats. 
